# Comment on: Men with delayed ejaculation report lower sexual satisfaction and more depressive symptoms than those with premature ejaculation: findings from a cross-sectional study

**DOI:** 10.1038/s41443-025-01173-9

**Published:** 2025-09-16

**Authors:** David L. Rowland

**Affiliations:** Department of Psychology, Valparaiso University, Valparaiso, IN USA

**Keywords:** Health care, Quality of life

The etiologies of delayed and/or inhibited ejaculation (DE) are both varied and poorly understood. Potential contributing factors include a predisposition to prolonged ejaculation latencies (EL) during partnered sexual activity; the erotic value tied to specific psychosexual and physical stimulus parameters (e.g., type of sexual activity, pornography, nature of fantasies, auto-erotic preference, etc.); relational dynamics such as partner dysfunction; and the role of sexual anxiety in precipitating, maintaining, or exacerbating the problem [1–3]. I commend Negri et al. in their recent study [4] for attempting to identify specific characteristics about this population, including risk factors and consequences related to the dysfunction. The finding that men with DE reported greater depressive and anxiety symptomology than men with PE is noteworthy, and as such identifies a possible avenue for consideration in the treatment of these men. However, as the authors themselves note, it is not known which factor is the culprit here—whether the depression/anxiety contributes to, or is the consequence of, the sexual problem.

However, to make comparisons across types of sexual dysfunctions, and to conclude that one type of dysfunction is associated with greater distress and/depression, raises many questions, including some regarding methodology. We, for example, have found paradoxical results with respect to the role of distress in men with DE. On the one hand, we have found that distress represents a much more salient factor in the diagnosis of DE than it does in the diagnosis of PE [5–7]. Yet, we have also found that levels of self-reported distress/anxiety in men with DE were significantly *lower* than those of men with PE [1, 8]: our hypothetical explanation—yet to be empirically tested—was that PE men—whose ejaculation occurs relatively quickly after penetration—would have greater difficulty satisfying their (female) sexual partner than men whose penetrative duration is sufficiently (and plentifully) long for the (female) sexual partner to reach orgasm [8–12]. In other words, men with DE do not bear the same level of “partner” burden as men with PE [13]. In those studies, we attempted to control for the men’s perceived severity of their dysfunction.

This brings us to the issue of methodology. In any quasi-experimental design where patients are not randomly assigned to groups, it is critically important to demonstrate that those groups are equivalent on any uncontrolled variables (e.g., age, comorbidities, etc.) that might be related to the outcome variables (in this case, anxiety and/or depression) (Note: although the title of the study suggests group differences between PE and DE men on sexual satisfaction, this difference did not emerge in the IIEF domains of intercourse satisfaction and overall satisfaction as presented in Table 1 of the original document). Furthermore, to arrive at the conclusion that men with DE are generally more depressed or anxious than men with PE requires a demonstration that the severity of symptomology for the two groups was near equivalent. However, demonstrating such equivalency would be, in and of itself, a challenge, given the authors’ criteria for diagnosing DE. Yet, if the two groups were not equivalent on this dimension, then the differences in anxiety/depression may not be due to the type of dysfunction but rather to the severity of the sexual problem [14].

The authors also demonstrated that a number of other parameters (i.e., besides anxiety and depression) differ between DE and PE men, including their age, the likelihood of having a comorbidity, and their level of sexual desire, all of which could account for the differences in anxiety/depression between the groups (independent of the type of dysfunction). Thus, a methodologically-better strategy for demonstrating (with any degree of confidence) that the differences in anxiety/depression are due to the specific dysfunction type, is to control for those “other” variables that differ across dysfunction types. Statistically, this is typically accomplished with a regression analysis where each variable can be entered as a covariate, the theoretical or practical importance of each covariate can be established a priori by the order of entry of the covariate, and the general effect size of each of the covariates can be gauged (roughly) by the beta coefficients. In addition, the collinearity of the covariates can be assessed, so those that are contributing significant overlapping variances can be identified and managed [15].

In fact, the authors had already collected valuable data relevant to their research question, so it was a disappointment that they did not use these variables and a statistical methodology that would have enabled them to draw stronger inferences about a potentially-unique relationship between dysfunction type and anxiety/depression—a missed opportunity indeed, as the authors could have begun to better identify factors that explained the “how” and “why” behind the differences that they have uncovered.

In conclusion, descriptive studies of the type reported by Negri et al. [4] represent important starting points for understanding men with DE. In doing so, these authors have uncovered a phenomenon that deserves further exploration, including not only studies that provide independent verification of the reliability of the effect, but also hypothesis-driven studies that identify factors that might account for the differences between DE and PE populations. For example, DE and PE men differ in their capacity to conceive children; in their levels of comorbidities; in their capacity to experience sexual pleasure; and along numerous other dimensions, any and all of which may contribute to differences in anxiety and depression between the two types of dysfunctions. My hope is that the authors will pursue follow-up analyses that offer a more detailed and comprehensive investigation into the phenomenon they have reported.
